# National Association of Dental Examiners

**Published:** 1895-10

**Authors:** 


### National Association of Dental Examiners.

The thirteenth annual session of the National Association of Dental Examiners was held at Asbury Park, N. J., commencing Monday, August 5, 1895; the president, Dr. L. Ashley Faught, of Philadelphia, in the chair.

The following state boards were represented at the sessions:

*Alabama*—T. P. Whitby.

*Delaware*—C. R. Jefferis, D. M. Hitch.

*Georgia*—J. H. Coyle.

*Iowa*—J. T. Abbott.

*Kentucky*—H. B. Tileston.

*Kansas*—J. O. Houx.

*Colorado*—R. B. Weiser.

*New Jersey*—F. C. Barlow, Chas. A. Meeker, Geo. E. Adams, E. M. Beesley.

*Pennsylvania*—Louis Jack, W. E. Magill, L. Ashley Faught, Jesse C Green.

*Tennessee*—F. A. Shotwell.

*Virginia*—J. Hall Moore.

*District of Columbia*—H. B. Noble, Williams Donnally.

The following boards were elected to membership:

*Connecticut*—Geo. L. Parmele.

*New York*—Wm. Carr.

*New Hampshire*—Edward B. Davis.

A resolution, offered by Dr. Barlow, requiring credentials to the association to bear the official seal of the state board making the application, was adopted.

A resolution offered by Dr. Donnally last year, and laid over, permitting persons who have been delegates to the association to be associate members without the right to vote or hold office, was taken up and adopted.

Dr. Jack offered the following, which was adopted.

*Resolved*, That this body would express to the Association of Faculties the importance of an examination of the equipment, methods and facilities of instruction of all the dental colleges of this country; it being understood that such examination is to be purely in the interest of higher educational standards and toward an approach to ultimate uniformity in the curriculum and methods of the schools, and more particularly to enable safe action to be made with respect to new schools.

Later a communication was received from the secretary of the National Association of Dental Faculties to the effect that the association had ordered the secretary to secure information from the various colleges regarding their equipment and general facilities for teaching; that this information would be systematized so as to be available at the next annual meeting of this body.

The following "plan of requirements for the recognition of dental schools," offered by Dr. Jack, was adopted, with a proviso that it shall apply only to colleges making application after the close of this session:

That each dental school which may in future come before this board for recognition, must have a teaching faculty composed as follows, to wit: at least three professors of dental subjects, namely, for operative dentistry, for dental prosthetics, for dental pathology and therapeutics. For the medical subjects there must be at least five professors, namely, for anatomy, for physiology, for chemistry, for pathology, and for materia medica.

Its students must also be taught the subjects of chemistry and bacteriology in laboratories adapted to the purpose and under suitable instructors.

That such special school must possess, in addition to suitable lecture-rooms, a well-appointed dental infirmary and a general prosthetic laboratory; also each school must be provided with a room or rooms suitable for manual training in operative dentistry, and must furnish in this way systematic instruction to its students.

All of these provisions are to be determined by careful inspection on the part of the Board of Examiners of the state within which is located the school, or other authorized body duly indorsed by this association. And upon the result of this examination may depend the question of reputability.

The following colleges were added to the list of recognized schools: Dental Department of the University of Denver, Denver, Col.; Department of Dentistry of Detroit College of Medicine, Detroit, Mich.; Dental Department of Western Reserve University, Cleveland, O.

Applications from the following were laid over one year: University of Buffalo, Dental Department; Atlanta Dental College; University College of Medicine, Dental Department, Richmond, Va.; Birmingham Dental College; Cincinnati College of Dental Surgery.

The Committee on Colleges in its report, which was presented by its chairman, Dr. Jack, expressed the view that more should be required to establish the right of dental schools to recognition by this body than good organization and the fulfillment of the rules of the Association of Faculties. Evidence should be furnished that the teachers are of high standing; that they require of their matriculates the stipulated preliminary training, and that they are carefully qualifying their students in every necessary direction. To ascertain these facts is a matter of difficulty. It is necessary, too, in addition to an ascertainment of the character of the faculties of any school, to discover the degree of confidence which has been developed in the minds of the local members of the profession.

The number of students in actual attendance in all the schools of the country for the session 1894–95, excluding those attending special courses, was 4979, as against 3997 at the previous session; graduates 1207, as against 911.

The committee also expressed the conviction that it is becoming evident that the dental schools are increasing in number beyond the needs of the public, owing to the tendency of medical schools to inaugurate dental departments. The installation of dental departments in connection with medical schools is necessarily often incomplete, and therefore the committee believes that restrictions should be placed upon the rapid increase of inefficient dental colleges. As the practice of dentistry is largely based upon knowledge of chemistry and bacteriology, and as manual training has become an integral part of the curriculum of some of the better schools, we recommend that the association do not in future recognize any school unless satisfactory evidence is furnished that the students of such schools applying for recognition are being taught in modern chemical and bacteriological laboratories, and are also furnished with every convenience for manual training in prosthetic and operative dentistry, and that this latter mode of practical instruction is systematically carried on in at least the first year's course.

The committee also called attention to the importance of a higher standard of preliminary education, and to the impropriety of schools advertising as instructors practitioners who occasionally clinic before the students, but are not a part of the staff of the institution.

The report was adopted.

The following resolution, offered by Dr. Magill, was unanimously adopted:

*Resolved*, That we will not in future consider favorably an application for recognition from any college which has as a member of its faculty one who also holds membership in the State Examining Board.

Dr. Donnally moved that final action shall not be taken on the application of any college until such application has been in the hands of the chairman of the Committee on Colleges for at least ten months so ordered.

The following were elected officers for the ensuing year: J. T. Abbott, Manchester, Iowa, president; H. B. Noble, Washington, D. C., vice-president; Charles A. Meeker, Newark, N. J., secretary and treasurer.

Adjourned.

